# Identification and Characterization of *Mycobacterium Tuberculosis* Isolates from Cattle Owners in North Western and North Eastern Parts of Rural Ethiopia

**Published:** 2015-01

**Authors:** Araya Mengistu, Fikre Enquselassie, Elena Hailu, Abraham Aseffa, Demissew Beyene

**Affiliations:** 1 Faculty of Veterinary Medicine, University of Gondar, Ethiopia; 2 School of Public Health, Addis Ababa University, Ethiopia; 3 Armauer Hansen Research Institute, Addis Ababa, Ethiopia

**Keywords:** Ethiopia, Lineages, Mycobacterium tuberculosis, SIT, Tuberculosis, Typing

## Abstract

Tuberculosis (TB) is a major global public health problem resulting in a considerable morbidity and mortality worldwide. Ethiopia ranks 8^th^ among the 22 high TB burden countries. Establishing an appropriate and improved intervention strategy to prevent and control tuberculosis requires the presence of evidence based data on the genetic diversity of its causative agent. The current research work, therefore, was to differentiate strains of *Mycobacterium tuberculosis* isolated from pulmonary tuberculosis patients who own cattle in North Eastern and North Western parts of Ethiopia using molecular techniques. Sputum samples were collected from Acid Fast Bacilli (AFB) positive pulmonary tuberculosis patients and cultured on Lowenstein-Jensen (LJ) medium containing glycerol and sodium pyruvate. Deoxyribonucleic acid (DNA) was extracted from each positive culture, spoligotyping and single nucleotide polymorphisms were performed to further differentiate strains of *M. tuberculosis*, after deletion typing PCR confirmed that all the isolates were *Mycobacterium tuberculosis*. The mean age of study participants was 35.7 years (18–63 years) + 13.24. The majority (55.7%) were from North Gondar zone. Spoligotyping revealed that (47/50) 94% had interpretable patterns and 3 lineages namely; East-Africa-Indian (57.4%), Euro-American-African (EAA lineage-Lineage 4) 38.3% and Ethiopian (lineage-7) 2/50 (4.3%). Lineage 7 was registered in North Wollo zone only. In this study 8 clusters (with cluster size ranging from 2–8), 8 unique and 10 new patters were recorded. Spoligotype International Types (SIT) (21, 25, 26, 35, 53, 109, 149 and 289), were found as clusters and of these SIT 25 (7) and SIT 289 (8) were the predominant ones. Our study proved that 3 *Mycobacterium tuberculosis* lineages, namely; the ancient, intermediate between the modern lineages as well as modern were identified. Besides, considerable clustering was seen, which indicates the presence of current TB transmission in the study areas.

## 1 Introduction

Tuberculosis (TB) as a disease traveled for several decades with humans [1], [2], [3] and yet remains as a major global public health problem [4] despite the availability of anti-TB drugs. In 2012 there were 8.6 million new TB cases and 1.3 million deaths [5]. According to WHO 2013 report, Ethiopia ranked 8^th^ among 22 TB high burden countries with an estimated 247 (183–321) (in thousand population) incident rate including HIV positives. According to Federal Ministry of Health (FMoH), Ethiopia, report TB is one of the leading causes of morbidity, the fourth main cause of hospital admission, and the second largest cause of hospital deaths (after malaria) [6] and TB is endemic in the Amhara region state as well. A study that has collected sputum samples from different health institutes in the region were cultured and among 240 positive cultures, 237 were *Mycobacterium tuberculosis* based on RD9 based PCR analysis [7]. Similarly, in South Wollo zone, Desie referral hospital (Neighbor to North Wollo zone, Woldeya) 6.2% smear positive cases were reported among diabetic patients [8]. Among 250 prisoners in North Gondar zone 26 (10.4%) were found to have tuberculosis [9]. The disease in humans is mainly caused by *Mycobacterium tuberculosis* and to some extent by other species of *Mycobacterium tuberculosis complex* [10], [11], [12], [13], [14]. Now day’s molecular technique has become a powerful tool and is widely used to type Mycobacterium tuberculosis complex species to know TB transmission dynamics [15], [16], [17], [18], [19]. Understanding TB epidemiology, determining either TB relapse or exogenous reinfection, outbreak investigation and knowing laboratory cross contaminations as well as identification of the spread of clones is possible using molecular genotyping [20], [21], [22], [23], [24], [25]. For the purpose of this study pulmonary tuberculosis patients who own cattle were considered to isolate *Mycobacterium tuberculosis complex species* from them and test their cattle for bovine tuberculosis. Therefore, the current research work aimed to look the genetic biodiversity of *Mycobacterium tuberculosis* isolates recovered from pulmonary tuberculosis patients who own cattle in North Eastern and North Western parts of Ethiopia using molecular techniques.

## 2 Ethical Considerations

Ethical clearance was obtained from the institutional review board (IRB) of the College of Health Sciences, Addis Ababa University and AHRI/ALERT ethics review committee (AAERC). Letter of support was obtained from the Amhara Regional Health Bureau and from North Gondar and North Wollo Zones as well as from the respective woreda health Bureaus. Written consent was obtained from each TB case study participants. Confidentiality was maintained by using codes instead of participants' names. Confirmed TB cases were referred to TB clinics and received all the necessary anti-TB treatments with counseling by professionals.

## 3 Methods And Materials

### 3.1 Study Area, Setting and Design

This study was conducted in North Gondar and Wollo zones of Amhara regional state, North Western and North Eastern Ethiopia, where the livelihood is mainly mixed farming. Dembia, Chilga, Dabat, Debark, Adarkay and Wegera from North Gondar zone and Meket, Gubalafto, Habru and RayaKobo Districts from North Wollo zone were included in the study. Based on 2007 Census, the study place covered an estimated area of 58,117.13 square kilometers with a total population of 4,429,931. Of these, the majority are rural dwellers accounted for about 90% and 85% in North Wollo and North Gondar zones, respectively [26]. Acid fast bacilli (AFB) positive sputum samples were collected cross sectional from August 2012–August 2013 from TB patients who own cattle at hospitals and health centers in both zones.

### 3.2 Study Populations

TB patients having cattle were identified at their respective health institute and used as a study population. A human TB case was defined as a smear positive adult pulmonary TB patient diagnosed at the respective health institutes in the study zones.

### 3.3 Sample Size

During the follow up study period, we encountered willing 70 AFB positive TB patients who own cattle in both zones. A questionnaire used to collect information and willingness to participate in the study was the main criteria to identify participants.

### 3.4 Sputum Sample Collection and Culturing

For *Mycobacterium tuberculosis* isolation sputum samples were collected from Acid Fast Bacilli (AFB) positive patients. The Sputum samples were collected according to Cheesbrough *et al*., [27]. Briefly, the aim of the study was explained to the patients who were active pulmonary tuberculosis cases with smear positive findings and his/her willingness was asked to participate in the study. If he/she is willing to participate, sputum samples were collected from the patient. Patients were asked to produce an “on spot” sputum specimen in a 20 ml screw plastic container under the supervision of a trained laboratory technician in an open air. If sputum was not produced within 15 minutes, the patient was excluded from the study. Those patients who produce sputum was asked to give additional sputum samples on the second and third day. The sputum samples obtained from each patient put in a cooler with ice packs (4°C) and immediately after collection it was transferred to the hospital laboratory to be stored in −20°c or refrigerator. The samples collected from each site put in a cooler with ice packs (4°C) to transport to the AHRI tuberculosis laboratory and then sputum samples were processed and cultured in the same laboratory.

Briefly, an equal amount of Phosphate-buffered saline (PBS) solution was added to the sputum and then was decontaminated, digested with equal volume of 4% sodium hydroxide for 15 minutes. Then vortaxing was done in a closed tube for 1–3 (5) minutes till the mixture becomes homogeneous. The homogenate was centrifuged at 3000 Revolution Per Minute (RPM) for 15 minutes. Neutralization was done by using concentrated hydrochloric acid. In order to monitor neutralization 1 or 2 drops of phenol red was added. The supernatant was decanted and the sediment was inoculated onto Lowenstein-Jensen medium (tubes containing glycerol and sodium pyruvate) using the drop method. The tubes were incubated at 37°C and examined for growth weekly for 12 weeks [28]. Cultures were considered negative if there is no mycobacteria growth after 12 weeks of incubation. Growth of mycobacteria was confirmed by detection of a typical colonial morphology and by microscopy for AFB after Ziehl-Neelsen staining. Positive cultures were sub-cultured onto another set of media and incubated for another 3–4 weeks for further identification. To characterize the isolates polymerase chain reaction (PCR) (deletion typing), spacer oligonucleotide typing (spoligotyping; which analyses polymorphism of direct repeat [(RD), RD patterns is important for typing systems for epidemiological and evolutionary studies of *M. tuberculosis*] region, a reliable technique and used to detect and type *M. tuberculosis*) were applied [29].

### 3.5 DNA Extraction

Briefly, a loop full of bacterial colonies were taken from culture positive Lowenstein-Jensen media and were transferred to the 1.5-ml tube containing 200 µl of 1% Tris-EDTA (TE) buffer. The re-suspended bacteria was heated in a dry bath at 90°C for 40 minutes and centrifuged at 10,000× *g* for 10 minutes. The supernatant was processed for target DNA isolation in the laboratory using the method described by Chakravorty and Tyagi [30].

### 3.6 PCR Using the Direct Repeat Deletion Technique

Differentiation of *Mycobacterium tuberculosis* complex species is made by PCR amplification of species-specific DNA fragments. Polymerase Chain Reaction (PCR), which encompasses denaturation, annealing and extension, was performed for all culture positive samples to identify the species of *the M. tuberculosis* complex causing tuberculosis in human using both RD4, RD9 and RD 10 regions as a marker with their respective specific forward, reverse and internal primers. The amplification products were analyzed by electrophoresis on 1.5% (W/V) agarose gel containing 1x ethidium bromide and were visualized with Ultraviolet rays [30]. To run the PCR-deletion typing the standard operation procedure used at AHRI, which is adopted from the Veterinary Laboratory agency (VLA), UK, was followed. Materials like Mycobacteria positive control strains: *M. tuberculosis H37RV* (ATCC 25681) and *M. bovis* AF2122/97 (ATCC BAA-935), ATCC (http:/WWW.lgcpromochem-atcc.com.), PCR Thermocycler, laminar flow cabinet, cleaning agent DNA away/equivalent), pipettes, filter tips, nuclease free water (Qiagen), HotStar Tagmaster Mix kit (Qiagen; Product catalog No 203445, which includes DNA polymerase, buffer, MgCl_2_, and dNTPS), stock solutions of oligonucleotide primers, namely; RD4-FlankFW, RD4-FlankRev, RD4-interanlFW, RD9-FlankFW, RD9-InternalRev, RD9-FalnkRev, RD10-FlankFW, RD10-InternalRev, RD10-FlankRev, heat killed cells that need to be detected and materials like Agarose gel electrophoresis equipments, DNA ladder and loading dye, agarose gel, 1XTAE running buffer and Ethidium bromide were used.

### 3.7 Spoligotyping

In order to further differentiate the *Mycobacterium tuberculosis* detected by PCR, spoligotyping (spacer oligonucleotide typing, Kamerbeek *et al*., [29] was performed by following the protocols used for AHRI spoligotyping. Spoligotyping, which used to detect and type bacteria of the genus *Mycobacterium tuberculosis* complex (MTC) includes three main methods, namely; PCR amplification of a specific spacer sequence of the strain, hybridization to a spoligomembarne and detection.

PCR was performed using DNA obtained from heat killed cells. The PCR amplification of the spacers was accomplished by using the primers RDa (5’-GGT TTT GGG TCT GAC GAC-3’) and RDb (5’ –CCG AGA GGG GAC GGA AAC-3’), which anneal to all repeat sequences and thereby enables for amplification of all spacers that occur in the DR region of the specific strain. The PCR products were labeled with biotin since the DRa primer was biotinylated. The PCR products amplified were loaded onto the membrane at right angle to the 43 parallel spacer lines by using a miniblotter and left for hybridization. After hybridization the membrane was washed with buffer to remove the non-hybridized and non-specific bound PCR products. The step was followed by incubation with conjugated streptavidin-peroxidase, of which streptavidin binds to the biotin labeled PCR products.

The presence or absence of spacers in specific strains was then detected by an enhanced chemiluminescence (ECL) detection system onto an autorad (photographic film). When the membrane was exposed to ECL a substrate for peroxidase, the autorad will detect light signals where hybridization has occurred and thereby produced a pattern that allows for spoligotyping of an isolate. Acceptable results include clearly defined squares on a clear background ensuring that the controls have worked, especially the negatives (water) control. Materials such as all reagents needed for PCR amplifications, baths, glass bottles, measuring cylinders, scales, magnetic stirrers, pH meter, thermocyclers, autorad and gelelectrophorosis as well as among the consumable stock solutions, buffer solutions (10% SDS, Sodium dodecyle sulphate), 0.5M EDTAX2H_2_0 and other chemicals/salts needed for hybridization and detection were utilized. In addition positive and negative controls and samples to be typed were used.

### 3.8 Single Nucleotide Polymorphisms (SNPs) Typing

After predicting the lineages of the isolates by Spoligotyping final confirmation for Lineage 3 and Lineage 4 was done by SNPs typing using real-time PCR. We performed the TaqMan Real-time PCR (Rotor Gene) SNP assay for lineage 4 (Euro-American, E-A lineage) and lineage 3. The lineage 4 was defined by katG 463 SNP and the assay was performed as described [31]. The lineage 3 was defined by Rv3804c_0012s SNP and assay was performed using standard procedures [32].

## 4 Results

The age of participants ranged from 18–63 years old with a mean age of 35.7 years ± 13.24. Of them, 45 (64.3%) were between 18–40 years. Majorities (55.7%) were from North Gondar zone with 1:1 male to female ratio. Among the participants, 64.3%, 82.9%, 41.5%, 60.0%, 38.6%, 20.0%, 52.9%, 38.6% and 71.4% were illiterate, rural dwellers, farmers, married, had contact with TB patients before, treated with anti-TB drugs before, took unboiled milk as well as uncooked meat and revealed culture positive results, respectively (Table-1).

Of the 70 AFB positive sputum samples cultured, 28/70 (40%) and 22/70 (31.4%) were culture positive from North Gondar and North Wollo zones, respectively. As shown in Table-1 among culture positives, 33/70 (47.1%), 17/70 (24.3%), 10/70 (14.3%), 26/70 (37.1%) and 23/70 (32.8%) were married, had previous contact with TB patients, received anti-TB treatment before, experienced drinking unboiled milk and consuming uncooked meat.

Seventy AFB positive sputum samples were collected in North East and North West Ethiopia from pulmonary tuberculosis patients. Sputum samples were collected from 8 different health institutes which are found in North Gondar and North Wollo zones. All 50 AFB positive samples were *Mycobacterium tuberculosis* based on deletion typing. Further, isolates characterizations were done using spoligotyping by comparison with the International SpoIDB4 database. Of these, ninety four percent (47/50) showed good interpretable patterns. Forty six point eight percent (22/47) and 53.2% (25/47) were from North Wollo and North Gondar zones, respectively.

**Figure-1:**
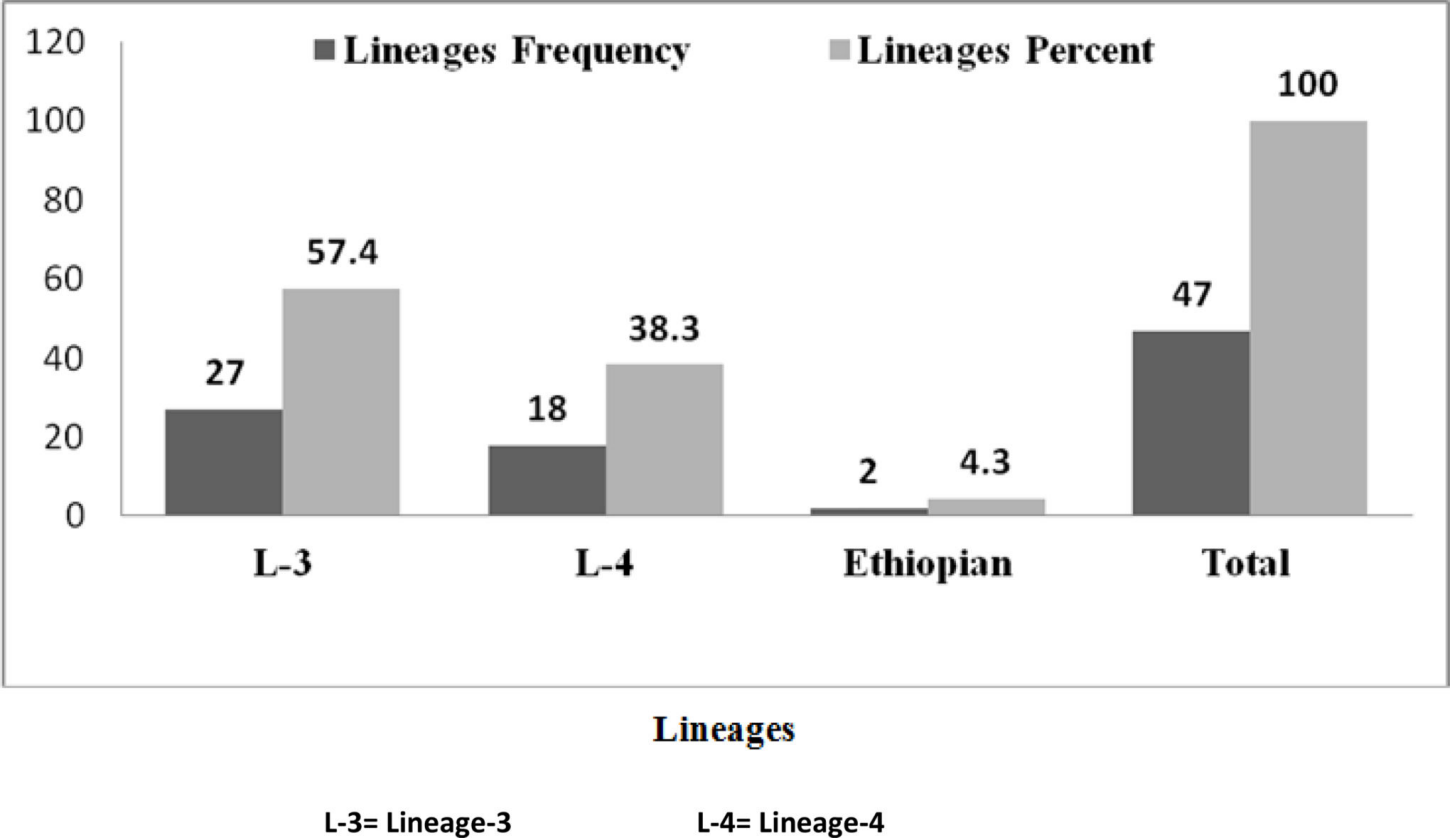
Lineages identified in the Northwest and Northeast part of Ethiopia, 2014

As it is presented in Figure-1, three lineages namely; lineage 3 (East African-Indian) 57.4% (27/47), 4 (Euro-American-African) 38.3% (18/47) and 7 (Ethiopian) 4.3% (2/47) were recorded in the study areas. Among lineage 3, 5 SITs and 6 new SITs, lineage 4, 9 SITs with 4 new SITs, and lineage 7, 2 SITs were identified in both areas. In general 16 shared international types with 10 new ones were disclosed by the current study. Among the SITs, SIT 25 (7), 26 (3) and 289 (8) were the predominant ones. From the new SITs 4 and 6 were from North Wollo and North Gondar zones, respectively. Based on spoligotyping 8 clusters (with a cluster size ranging from 2–8), 8 unique and 10 new patterns were identified in the study areas. SITs (21, 25, 26, 35, 53, 109, 149 and 289) was found as clusters in both zones. From 3 cluster size/isolates of SIT 53, 66.7% (2/3) and SIT 26 with cluster size of 3, 66.7% (2/3) were from North Wollo zone. SIT 149 with cluster size 2 and SIT 109 with cluster size 2 were noticed from North Wollo zone only. SIT 35 and SIT 21 each with cluster size 2 reported in North Gondar zone, particularly the first one registered in Chilga Woreda. Among SIT 25 with cluster size 7, 57.1% (4/7) and SIT 289 with cluster size 8, 75% (6/8) occurred in Debark Woreda areas and Chilga Woreda of North Gondar zone, respectively (Table-2). The rest was identified as unique which may indicate reactivation of an old infection, or imported TB or may result from missing isolates.

## 5 Discussion

Tuberculosis in the rural community is higher compared to the urban settings and its occurrence could vary depending on the awareness of the community. In the current study, besides the community habits of drinking unboiled milk and eating uncooked meat, nearly 83% and 64.3% were living in the rural community and illiterate. The aforementioned factors could facilitate the transmission of different lineages of *Mycobacterium tuberculosis* among the community members or households. In this study those leading school lives (primary, secondary and college) contributed about 22.9% (16/70) for culture positivity, which could serve as a good medium for TB transmission due to the possible chance of the congregations [33], [34]. We recorded that nearly a quarter of TB cases had contact, which considered as previous contact history as strong factor for active TB development [35], [36], [37], with TB patients before their sickness.

Among the tools, genotyping is contributing a lot to understand the epidemiology of TB and provides information on its transmission dynamics [20]. Based on deletion typing 50 were found to be *Mycobacterium tuberculosis*. In this study, 78.7% of SITs were matched with the standard spoligotype database while 21.3% were new ones. The current study revealed the presence of 3 lineages in North West and North East Ethiopia. The majority (57.4%) were the East African-Indian lineages (EAI; L-3) followed by Euro-American-African (EAA; L-4) (38.3%) lineages, which were found in both zones while Ethiopia (L-7) (4.3%) were found in North Wollo zone only. Since EAI strains reported as one of the major genotypes in many countries of Southeast Asia and Africa [38], the higher proportion of this lineage finding in the current study could agree with the stated assumption. In contrast to the current finding, fewer value of EAI lineages were reported in a study conducted from farmers in mixed type multipurpose cattle raising region of Ethiopia, the majority 78.5% were Euro-American lineage followed by East-African-Indian, 17.7% [39] and Rebuma and his colleagues also reported the presence of these lineages in both zones and stated 71% (442/662) and 25% (153/622) lineage 4 and 3 from PTB patients, respectively, and 70% (229/328) and 24% (79/328) lineage 4 and 3 from TB lymphadenitis (TBLN) patients, respectively [40]. The Authors also reported relatively a higher percentage that is 13% (17/133) of a new lineage, named as lineage-7 [40], which placed between ancient lineage 1 and modern lineages of *Mycobacterium tuberculosis* phylogeny prominently in North Wollo (woldiya) area compared to the current study. The difference in the proportion could be due to sample size in the case of North Wollo zone and geographical situations in the case of central Ethiopia. In Uganda, researchers reported 22% (394/1746) and 11% (187/1746) L-4 and 3, respectively [41]. The current finding showed relatively higher values compared to the work in Uganda and this might be due to the presence of dominant and common types of prevailing isolates differences in both countries. Lineage East African-Indian showed higher cluster size by SIT 25 (7 cluster size) and 289 (8 cluster size).

Although 3 *Mycobacterium tuberculosis* lineages recorded in this study, failure of isolation of any of *Mycobacterium tuberculosis complex species* from skin test positive cattle should be seen as a limitation of this study, since the aim of the study was to see the possible role of cattle in the epidemiology of human tuberculosis by determining the presence of cross infections with detecting the same strain in the same house.

## 6 Conclusion

It is concluded that 3 *Mycobacterium tuberculosis* lineages, namely; the ancient, intermediate between the modern lineages and modern were identified in the study areas. Among the isolates, 61.7 % (29/47), (including the new isolates to the denominator) were clustered indicating the presence of recent tuberculosis transmission in both areas. Health institutes need to work more to mitigate TB transmission among the community. Early disease detection and treatment of active TB cases timely as well as effectively will definitely curve TB incidence as well as prevalence. The reasons for the restricted occurrence of Lineage 7 in the area, including socio-cultural determinants should be investigated.

## Figures and Tables

**Table 1:** Sociodemographic characteristics of AFB positive TB patients and sputum culture result in Northwest and Northeast Ethiopia, 2014.

N=70				

			Culture result	
Category	Frequency	Percent	Negative	Positive
**Zone**				
North Gondar	39	55.7	11(15.7)	28(40.0)
North Wollo	31	44.3	9(12.9)	22(31.4)
**Sex**				
Male	35	50	13(18.6)	22(31.4)
Female	35	50	7(10.0)	28(40.0)
**Educational status**				
Illiterate	45	64.3	11(15.7)	34(48.6)
Primary school	13	18.6	6(8.6)	7(10.0)
Secondary school	6	8.6	1(1.5)	5(7.2)
College	4	5.7	2(2.8)	2(2.8)
Informal	2	2.8	0	2(2.8)
**Dwelling**				
Urban	12	17.1	4(5.7)	8(11.4)
Rural	58	82.9	16(22.9)	42(60.0)
**Occupation**				
Merchant	5	7.2	2(2.9)	3(4.3)
Student	14	20.0	7(10.0)	7(10.0)
Housewife	19	27.1	5(7.1)	14(20.0)
Government employee	3	4.3	0(0)	3(4.3)
Farmer	29	41.5	6(8.6)	23(32.8)
**Marital status**				
Single	19	27.1	7(10.0)	12(17.1)
Married	42	60.0	9(12.9)	33(47.1)
Divorced	5	7.2	2(2.8)	3(4.3)
Widowed	3	4.3	2(2.8)	1(1.5)
With parents	1	1.4	0(0)	1(1.5)
**History of contact with TB patients**				
No	43	61.4	10(14.3)	33(47.1)
Yes	27	38.6	10(14.3)	17(24.3)
**Anti-TB treatment before**				
No	56	80.0	16(22.9)	40(57.1)
Yes	14	20.0	4(5.7)	10(14.3)
**Taking unboiled milk habit**				
No	33	47.1	9(12.9)	24(34.3)
Yes	37	52.9	11(15.7)	26(37.1)
**Taking uncooked meat habit**				
No	43	61.4	15(21.4)	28(40.0)
Yes	27	38.6	5(7.1)	22(31.5)
**Sputum culture result**				
Negative	20	28.6		
Positive	50	71.4		

Numbers in parenthesis are percentages.

**Table-2:** Description of shared International types (SITs) representing M. tuberculosis isolates from AFB positive sputum samples from cattle owners in North East and North West Ethiopia, 2014

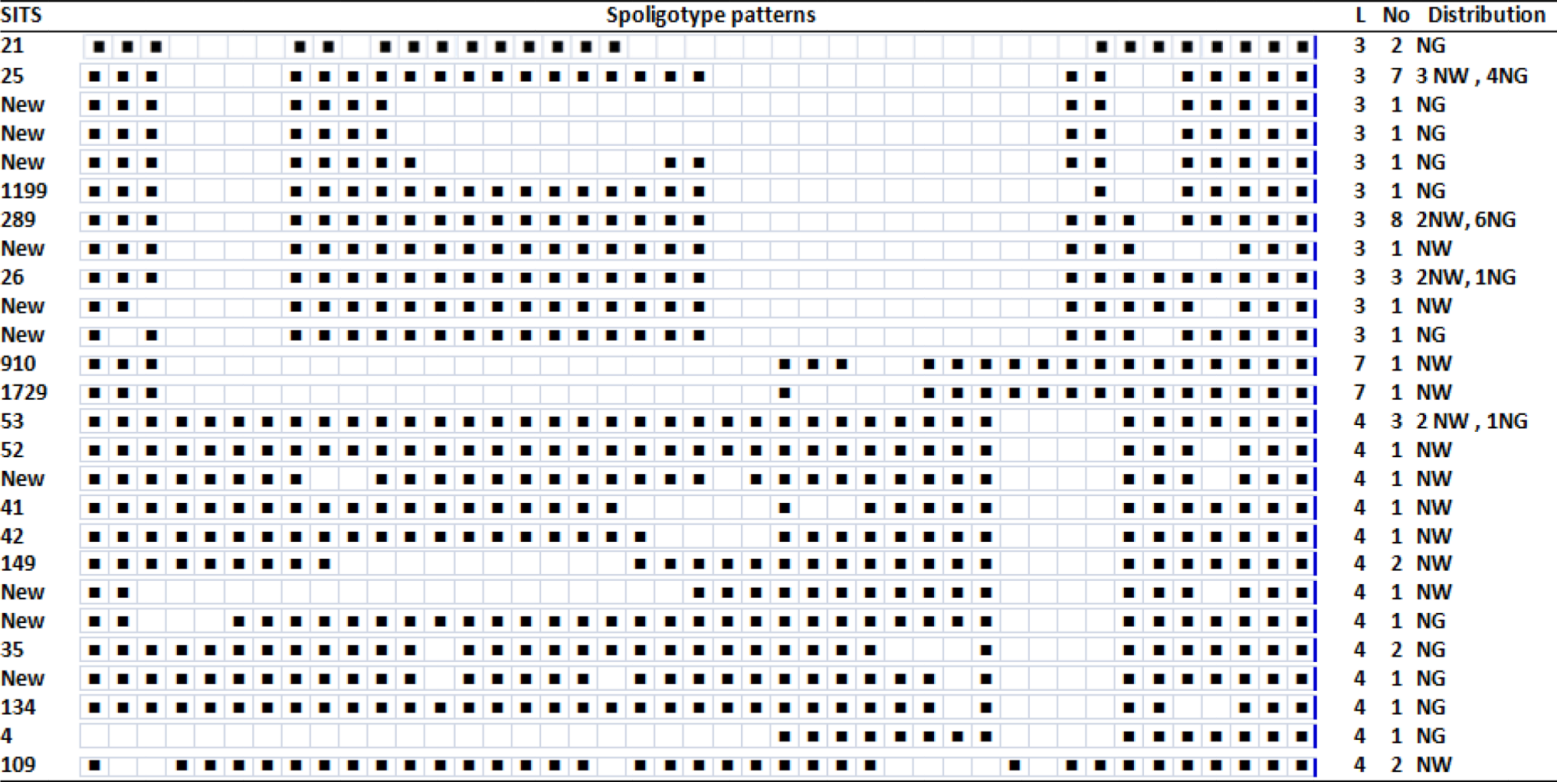

SITs: Spoligotypes/Shard International types L: Lineage NW: North Wollo NG: North Gondar No: No of isolates Black boxes: Interpretable patterns (represents the presence of the specific spacers at position) White boxes: Not interpretable patterns (represents absence of the specific spacers at positions 1–43 in the Direct Repeat (RD) Locus.
